# Characterization of CRH-Binding Protein (CRHBP) in Chickens: Molecular Cloning, Tissue Distribution and Investigation of Its Role as a Negative Feedback Regulator within the Hypothalamus–Pituitary–Adrenal Axis

**DOI:** 10.3390/genes13101680

**Published:** 2022-09-20

**Authors:** Yiping Wan, Zheng Zhang, Dongliang Lin, Xinglong Wang, Tianjiao Huang, Jiancheng Su, Jiannan Zhang, Juan Li, Yajun Wang

**Affiliations:** 1 Key Laboratory of Bio-Resources and Eco-Environment of Ministry of Education, College of Life Sciences, Sichuan University, Chengdu 610065, China; 2 Laboratory of Liver Surgery, West China Hospital, Sichuan University, Chengdu 610065, China

**Keywords:** chicken, CRHBP, ACTH, hypothalamus, pituitary, stress

## Abstract

Corticotropin (ACTH) is a pituitary hormone playing important roles in stress response within the hypothalamus–pituitary–adrenal (HPA) axis. The biosynthesis and secretion of ACTH are controlled by multiple factors, including corticotropin-releasing hormone (CRH). As a key hypothalamus-derived regulator, CRH binds to corticotropin-releasing hormone receptor 1 (CRHR1) in the anterior pituitary gland to regulate ACTH synthesis and release. Thus, CRH-binding protein (CRHBP), which binds CRH with high affinity to inhibit CRH-induced ACTH secretion from pituitary cells, draws wide attention. In contrast to the extensive investigation of CRHBP in mammals and other lower vertebrates, the gene structure, tissue expression and physiological functions of CRHBP in birds remain largely unknown. In the present study, using chicken (c-) as our animal model, we examined the gene structure, tissue expression and functionality of CRHBP. Our results showed that: (1) *cCRHBP* cDNA encodes a 345 amino acid precursor, which shares high sequence identity with that of mammals, reptiles, frogs and fish; (2) *cCRHBP* is abundantly expressed in the brain (cerebrum and hypothalamus), pituitary and ovary; (3) cCRHBP inhibits the signaling of cCRHRs induced by cCRH, thus reducing the cCRH-induced ACTH secretion from cultured chick pituitary cells; (4) stress mediators (e.g., glucocorticoids) and stress significantly upregulate *CRHBP* mRNA expression in chickens, supporting its role as a negative feedback regulator in the HPA axis. The present study enriches our understanding of the conserved roles of CRHBP across vertebrates. In addition, chicken is an important poultry animal with multiple economic traits which are tightly controlled by the HPA axis. The characterization of the chicken *CRHBP* gene helps to reveal the molecular basis of the chicken HPA axis and is thus beneficial to the poultry industry.

## 1. Introduction

In vertebrates, corticotropin (ACTH), which is released from the anterior pituitary gland, plays important roles in stress response and other physiological activities, including steroid hormone biosynthesis and adrenal development [1,2,3]. As the critical part of the hypothalamus–pituitary–adrenal (HPA) axis, the biosynthesis and secretion of ACTH are controlled by both hypothalamic stimulatory factors and inhibitory factors (e.g., CRIF) [4,5,6]. For example, neurons in the paraventricular nucleus (PVN) of the hypothalamus synthesize and secrete corticotropin-releasing hormone (CRH), which binds corticotropin-releasing hormone receptor 1 (CRHR1), expressed in the anterior pituitary corticotroph to regulate the ACTH synthesis and release [7,8,9]. The neuropeptide W (NPW), recently identified in chickens, which binds to the neuropeptide B/W receptor 2 (NPBWR2), inhibits ACTH synthesis and release [10].

CRHBP, a 37 kDa secreted glycoprotein, is reported to be actively involved in ACTH release regulation [11,12]. Studies have shown that CRHBP can bind CRH with nanomolar (IC_50_ = 0.54 nM) affinity [13,14]. Using luciferase assays, CRHBP inhibits CRH-mediated CRHR1 activation in HEK293T cells [15]. Moreover, CRHBP also attenuates CRH-induced ACTH release from cultured rat anterior pituitary cells [15,16,17] and mouse pituitary AtT-20 cell line [18]. Compared to the wildtype mouse, the mouse with *CRHBP* overexpression in the anterior pituitary shows the increase in hypothalamic *CRH* using in situ hybridization [19,20]. In the mouse with *CRHBP* deficiency, a significant loss in body weight and an increase in anxiety-like behavior have been detected, thus supporting that CRHBP is a negative regulator in CRH signaling [21].

The full-length cDNAs encoding *CRHBP* are originally cloned from the human liver and rat brain [16]. They all encode a 322 amino acid precursor, including an N-terminal signal peptide of 23 amino acids, an N-linked glycosylation site and ten conserved cysteines, which form five consecutive disulfide bonds [16]. Subsequently, the *CRHBP* gene is further identified in other vertebrate species, including mice [18], sheep [22] and *Xenopus laevis* [23]. Using in situ hybridization, *CRHBP* is found to be expressed in the central nervous system (CNS), including the prefrontal cortex [24], central and basolateral amygdala [25], the bed nucleus of the stria terminalis [26], ventral tegmental area [27] and various hypothalamic regions [28]. In addition to the CNS, *CRHBP* is also highly expressed in the pituitaries of rats [29] and mice [30].

In contrast to the detailed and extensive studies in mammals and lower vertebrates, the information of *CRHBP* gene remains unknown in birds. In chickens, the cDNA sequence and gene structure of *CRHBP* have been predicted in the Ensembl database; however, its sequence feature and tissue expression need further confirmation. In addition, as a negative regulator in the HPA axis reported in mammals, whether CRHBP plays similar role in chickens needs to be investigated. Therefore, using chicken as the animal model, our present study aims to: (1) clone and analyze the gene structure and sequence characteristics of chicken (c-) *CRHBP*; (2) investigate the tissue expression of *cCRHBP*; (3) examine the effect of cCRHBP on the signaling of cCRHRs induced by cCRH; (4) examine the effect of cCRHBP on ACTH secretion induced by cCRH in anterior pituitary cells; (5) examine the effect of glucocorticoids and stress on the *cCRHBP* mRNA expression in chickens. The present study, for the first time, characterizes the chicken *CRHBP* gene, thus enriching our understanding of its conserved roles across vertebrates.

## 2. Materials and Methods

### 2.1. Ethics Statement

All animal experiments were conducted in accordance with the Guidelines for experimental animals issued by the Ministry of Science and Technology of the People’s Republic of China. The experimental protocol performed in this study was approved by the Animal Ethics Committee of the College of Life Sciences, Sichuan University, China, and the assurance number is 20210308008 (8 March 2021).

### 2.2. Chemicals, Primers, Peptides, Antibodies and Animals

All chemicals were purchased from Sigma-Aldrich (St. Louis, MO, USA), and restriction enzymes were obtained from TaKaRa (Dalian, China). Dexamethasone (A2324) was purchased from APExBIO (Houston, TX, USA). Dexamethasone 21-phosphate disodium salt (D807084) was purchased from Macklin (Shanghai, China). All primers used in this study were synthesized by Youkang Biotechnology (Chengdu, China) and are listed in Table 1. Chicken (c-) CRH peptide (41 amino acids, SEEPPISLDLTFHLLREVLEMARAEQLAQQAHSNRKLMEII) with the amidated C-terminus was synthesized using solid-phase Fmoc chemistry (GL Biochem, Shanghai, China), dissolved to 100 μM in Dulbecco’s Modified Eagle’s Medium (DMEM, Hyclone, Logan, UT, USA) and stored at −80 °C. The purity of the synthesized peptide was greater than 95% (analyzed by HPLC) and its structure was verified by mass spectrometry. Rabbit anti-ACTH polyclonal antibody (ab74976) was purchased from Abcam (Cambridge, UK). Mouse anti-His monoclonal antibody (HT501) was purchased from TransGen Biotech (Beijing, China). Rabbit anti-β-actin monoclonal antibody (#4970) was purchased from Cell Signaling Technology (Danvers, MA, USA). All chickens (Lohmann layer) employed in the present study, including adult chickens (1 year old) and chicks (3 weeks old), were purchased from a local commercial company in Chengdu.

### 2.3. Total RNA Extraction, Reverse Transcription and Quantitative Real-Time PCR Assay

Six adult chickens (3 males and 3 females) were sacrificed, and various tissues were collected, including the cerebrum, midbrain, cerebellum, hindbrain, hypothalamus, spinal cord, anterior pituitary, heart, liver, cecum, rectum, testis and ovary. All tissue samples were stored at −80 °C for RNA extraction.

According to the manufacturer’s instructions, total RNA was extracted from chicken tissues using RNAzol reagent (Molecular Research Center, Cincinnati, OH, USA) and dissolved in diethylpyrocarbonate (DEPC)-treated water. Total RNA was reverse transcribed into cDNA using Moloney murine leukemia virus (MMLV) reverse transcriptase (Takara, Dalian, China) according to our previous study [31]. Firstly, in a volume of 5 μL, 2 μg of total RNA and 0.5 μg of oligo-deoxythymidine were mixed, heated for 10 min at 70 °C and chilled for 2 min at 4 °C. Secondly, in a reaction mixture with a total volume of 10 μL, the first strand buffer, 0.5 mM of each deoxynucleotide triphosphate and 100 U of MMLV reverse transcriptase were added. Reverse transcription (RT) was performed at 42 °C for 90 min.

The quantitative real-time PCR (qPCR) experiment was used to investigate the mRNA expression of *cCRHBP* according to our previously established method [32]. The qPCR primers of the chicken *CRHBP* were designed based on the amplified sequence of *Gallus gallus* (OP259501) using the Primer-BLAST tool at NCBI, as listed in Table 1. The primers (10 μM), dNTP (10 mM), Easy Taq Buffer, Easy Taq DNA polymerase (TransGen Biotech), Eva Green (Biotium), MilliQ-H_2_O and templates were mixed in a total volume of 20 μL. Then, the reaction mix was conducted on the CFX96 Real-time PCR Detection System (Bio-Rad). The amplification conditions included an initial denaturation for 10 min at 94 °C followed by 20 s denaturation at 94 °C, 15 s annealing at 60 °C and 30 s extension at 72 °C for 40 cycles. To assess the specificity of qPCR amplification, a conventional PCR reaction and gel electrophoresis were performed before the qPCR, followed by melting curve analysis after the qPCR. The relative mRNA levels of *cCRHBP* in the chicken tissues were calculated using the comparative CT quantification (2^−ΔΔCT^) method of qPCR. Moreover, the mRNA levels of *cCRHBP* were normalized by that of *β-actin* and then expressed as the fold difference compared with that of the midbrain.

### 2.4. Cloning, Sequence Alignment and Synteny Analysis of Chicken CRHBP

Based on the predicted sequences of the chicken *CRHBP* deposited in Ensembl Database (ENSGALT00010027397.1), gene-specific primers were designed to amplify the cDNA containing a complete open reading frame (ORF) of the *cCRHBP* from the chicken brain using RT-PCR. The amplified PCR product of *cCRHBP* was cloned into the pTA2 vector (TOYOBO, Osaka, Japan) and sequenced (Youkang, Chengdu).

We also searched for protein sequences of CRHBP in several vertebrates, including Japanese quail (XP_015704133), zebra finch (XP_002190680), human (NP_001873), mouse (NP_940800), sheep (NP_001009339), Chinese soft-shelled turtle (XP_006119190), *Xenopus tropicalis* (XP_002940913) and spotted gar (XP_006626779). The amino acid sequence of cCRHBP was aligned with that of other vertebrates using the ClustalW program (BioEdit). To determine whether the cloned chicken *CRHBP* is orthologous to *CRHBP* identified in other vertebrate species, the neighboring genes of *CRHBP* in genomic regions of chicken and other vertebrate species were examined, which was formatted using a genome browser Genomicus, available online at https://www.genomicus.bio.ens.psl.eu/genomicus-93.01/cgi-bin/search.pl (accessed on 16 January 2022) [33].

### 2.5. Tissue Expression Analysis of Chicken CRHBP Using RNA-seq Data

In addition to the qPCR, RNA-seq datasets were also employed for the investigation of the *CRHBP* tissue expression in chickens. One RNA-seq dataset from red jungle fowl (SRP016501) was established by Burge et al. in an effort to reveal the evolutionary dynamics of gene regulation across vertebrates [34]. The other RNA-seq dataset, also from the red jungle fowl (E-MTAB-6769), was obtained from seven organs (cerebrum, cerebellum, heart, kidney, liver, ovary and testis) throughout developmental stages from early organogenesis to adulthood [35]. In the present study, based on the RNA-seq databases, we quantified the gene expression level using the transcript quantitative analysis tool Salmon v0.10.2 and default parameters. The relative abundance of chicken *CRHBP* transcripts was expressed as the transcripts per million (TPM).

### 2.6. Generation of Chicken CRHBP Conditioned Medium

In the present study, human embryonic kidney (HEK) 293 cells were used to generate the chicken CRHBP-conditioned medium (cCRHBP_CM_). The full-length coding sequence of the chicken *CRHBP* (including a His-tag fused at the C-terminus) was first cloned into a pcDNA3.1(+) expression vector and sequenced by Youkang Biotechnology (Chengdu, China). Then, HEK293 cells were cultured in DMEM supplemented with 10% (*v*/*v*) fetal bovine serum (HyClone, Chengdu), 100 U/mL penicillin G and 100 mg/mL streptomycin in a 90 mm culture dish (Nunc, Rochester, NY, USA) and incubated at 37 °C with 5% CO_2_. A day before transfection, cells were seeded into 6-well plates at a density of 3 × 10^5^ cells per well. The cells were then transfected with a mixture containing 1000 ng cCRHBP–His expression plasmid (or an empty pcDNA3.1 vector as a negative control) and 2 μL of jetPRIME (Polyplus transfection, Illkirch, France) in 200 μL transfection buffer. After 24 h of transfection, serum-free medium was substituted for the original medium. The conditional medium (cCRHBP_CM_ and pcDNA_CM_) was harvested after 24 h and subjected for concentration through ultrafiltration spin.

Chicken CRHBP protein in the cCRHBP_CM_ was detected by western blot. Briefly, the concentrated conditional media were separated on 15% SDS-PAGE gels (Yamei, Shanghai) and transferred to a polyvinylidene difluoride (PVDF) membrane (Sigma-Aldrich, USA). The membrane was incubated in 5% nonfat dry milk (TBST solution) for two hours at room temperature to lessen nonspecific binding. Next, the membrane was washed three times with TBST, followed by incubation with the anti-His primary antibody (1:2000) for the duration of the night at 4 °C. After washing three times with TBST, the membrane was incubated with HRP-conjugated goat anti-mouse secondary antibody (1:5000, A9044, Sigma) for two hours at room temperature. Finally, blots were detected using an ECL chemiluminescence detection kit (Thermo Fisher Scientific, Waltham, MA, USA).

### 2.7. Effect of Chicken CRHBP on the Signaling of CRHRs Induced by cCRH in Chinese Hamster Ovary (CHO) Cells

In the present study, using the pGL3–CRE–luciferase receptor system, which was capable of the monitoring receptor-stimulated cAMP-PKA signaling pathway, we determined the effect of CRHBP on the signaling of cCRHRs activated by cCRH in CHO cells.

In chickens, two types of CRH receptors, *cCRHR1* and *cCRHR2*, have been cloned [36]. Based on the methods described in our previous studies [31,37], the signaling assays of chicken CRHRs (cCRHR1, cCRHR2) were first performed in CHO cells. Briefly, CHO cells were cultured in DMEM supplemented with 10% (*v*/*v*) fetal bovine serum, 100 U/mL penicillin G and 100 g/mL streptomycin in a 90 mm culture dish and incubated at 37 °C with 5% CO_2_. The CHO cells were seeded into 6-well plates one day before transfection. The cells were then transiently cotransfected with a receptor expression plasmid (cCRHR1 or cCRHR2) and a pGL3–CRE–luciferase reporter construct. The transfection mixture contained 250 ng of receptor expression plasmid, 750 ng of luciferase reporter construct, 2 μL of jetPRIME and 200 μL transfection buffer. After 24 h, the cells were subcultured on 96-well plates for an additional 24 h prior to treatment with CRH and CRHBP. The following are the processing steps for CRH and CRHBP.

For the CRH treatment, the cells were treated with 100 μL DMEM containing the desired dosages of cCRH (10^−12^ to 10^−6^ M) for 6 h. Finally, CHO cells were lysed using cell culture lysis buffer (Promega, Madison, WI, USA). According to the manufacturer’s instructions, the luciferase activity of the cell lysate was measured using a multimode microplate reader (TriStar LB941, Berthold Technologies, Bad Wildbad, Germany).

For the CRHBP treatment, the cells cotransfected with cCRHR1 (or cCRHR2) and pGL3–CRE–luciferase reporter constructs were exposed to a fixed dose of cCRH (1 nM) in combination with increasing amounts of cCRHBP_CM_ (0–20 μL added, total volume 60 μL/well). The pcDNA_CM_ (0–20 μL added, total volume 60 μL/well) was added to be employed as a negative control in the present study. After 6 h, the luciferase activity of CHO cell lysates was measured. The luciferase activity was represented as a percentage of the group treated with 1 nM CRH.

### 2.8. Effect of Chicken CRHBP on the ACTH Secretion Induced by CRH in Cultured Chick Pituitary Cells

According to our previously established method [38,39], anterior pituitaries were isolated and subjected for the primary culture from 3-week-old chicks. Pituitaries were digested by 0.25% trypsin at 37 °C for 30 min under sterile conditions. Dispersed pituitary cells were cultured at a density of 5 × 10^5^ cells/well in the 48-well plates (Corning, Tewksbury, MA, USA) with medium 199 (M199, Gibco, Thermo Fisher Scientific, Waltham, MA, USA) containing 15% fetal bovine serum and incubated at 37 °C with 5% CO_2_. After 18 h of culture, the cells were treated with cCRHBP_CM_ (20 μL added, total volume 120 μL/well) in the absence or presence of cCRH (5 nM) for 4 h. The conditional medium was then harvested for ACTH measurement through Western blot. Meanwhile, pituitary cells were lysed in RIPA buffer (100 μL/well) to examine the expression level of β-actin as an internal control.

### 2.9. Effect of Dexamethasone (DEX) on CRHBP mRNA Expression in Chickens

In the present study, in vitro and in vivo studies were further designed to examine whether glucocorticoids regulate the expression of *CRHBP* in the hypothalamus and pituitary in an effort to determine its role in the HPA axis.

Firstly, for the in vitro studies, pituitary cells were treated with various concentrations of DEX (0 nM, 1 nM, 10 nM and 100 nM) for 4 or 24 h. The total RNA was extracted from cultured pituitary cells and prepared for *CRHBP* mRNA expression analyses using qPCR.

For the in vivo studies, according to our earlier study [10], DEX was administered subcutaneously, and the hypothalamus and pituitary were collected for RNA extraction to determine the *CRHBP* mRNA expression using qPCR. In the experiment, three-week-old male chicks were reared at 22 °C with a 16L:8D (16 h light, 8 h dark) photoperiod and were fed with commercial diet regularly and water freely. Similar-weight chicks (~300 g/chick) were divided into two groups (*n* = 6): the control group (saline) and the experimental group (DEX). For adaptive training, a drug-free syringe needle was plunged subcutaneously into every chick’s abdomen at 9 a.m. every day for seven consecutive days. For the experimental group, the chicks were treated by a single subcutaneous injection of DEX (4 mg/kg body weight) in a 500 μL volume. For the control group, the chicks were subcutaneously injected with an equal volume of saline. Chicks were sacrificed three hours after injection, and hypothalamus and pituitary tissues were collected for RNA extraction. Simultaneously, all plasma samples were collected and subjected for ACTH (RXJ600533CH) and corticosterone (RXJ600484CH) concentration based on ELISA kits purchased from Ruixin Biotechnology company (Quanzhou, China).

### 2.10. Effect of Stress on CRHBP mRNA Expression in Chickens

In vertebrates, the HPA axis is actively employed for stress responses [5]. In the present study, in an effort to identify the CRHBP as a regulator in the HPA axis involved in stress response, its mRNA expression in the hypothalamus and pituitary were examined in chicks with stress treatments. The similar-weight male chicks (3-week-old) were first divided into two groups (*n* = 6): the experimental group and the control group. Three stress treatments were employed. For stress treatment 1: fasting for 24 h (the chicks were fed water freely but deprived of food for 24 h versus chicks which were fed food and water freely). For stress treatment 2: fasting for 48 h (the chicks were fed water freely but deprived of food for 48 h versus chicks which were fed food and water freely). For stress treatment 3: restraint for 1 h (the chicks were placed in a homemade cage (15 × 15 × 20 cm) with restricted mobility for 1 h versus chicks which were unrestrained). After the stress treatment, the chicks were sacrificed, and their hypothalamus and pituitary tissues were collected for RNA extraction. Following that, the *CRHBP* mRNA expression were examined using qPCR assay. In the present study, ACTH (RXJ600533CH) and corticosterone (RXJ600484CH) concentrations in all plasma samples were measured based on ELISA kits purchased from Ruixin Biotechnology company (Quanzhou, China).

### 2.11. Data Analysis

The protein bands of Western blotting were quantitated using densitometric analysis (ImageJ 1.52a, National Institutes of Health, Bethesda, MD, USA). The relative ACTH protein levels normalized by intracellular β-actin were represented as a percentage compared with the control group. The luciferase activities were represented as a percentage of the group treated with 1 nM CRH. The relative mRNA levels of *cCRHBP* in pituitary cells were first calculated as ratios to β-actin and then expressed as a percentage compared to their respective controls. The data were analyzed by one-way ANOVA followed by the Dunnett’s test in GraphPad Prism 8 (GraphPad Software, San Diego, CA, USA). To validate our results, all experiments were repeated twice or thrice.

## 3. Results

### 3.1. Cloning, Sequence Alignment and Synteny Analysis of Chicken CRHBP

**Figure 1:**
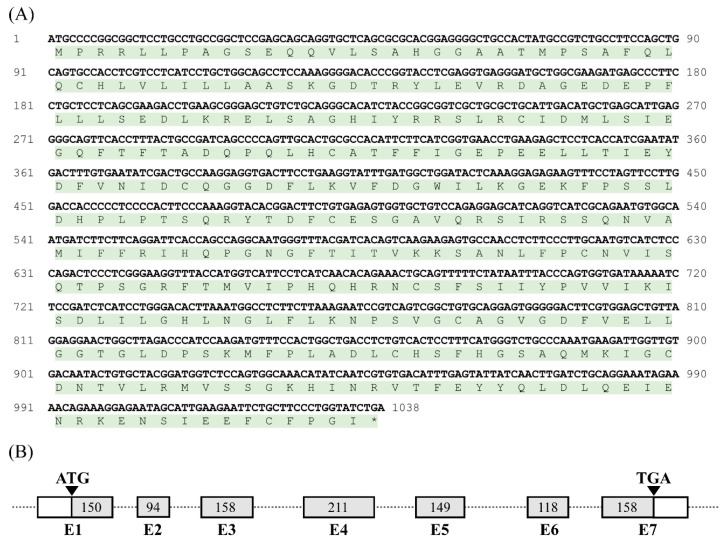
(**A**) The cDNA and deduced amino acid sequence of chicken *CRHBP*. The amino acids are shaded in green. The asterisk (*) indicates the stop codon. (**B**) Exon (E)-intron organization of chicken *CRHBP*. *cCRHBP* consists of 7 exons (E1–E7). The translation start (ATG) and end (TGA) sites are shown by arrows. The coding region of c*CRHBP* is shaded. The numbers in the boxes indicate the sizes (bp) of coding exons. Exons 1–7 are represented by the letters E1–E7.

**Figure 2:**
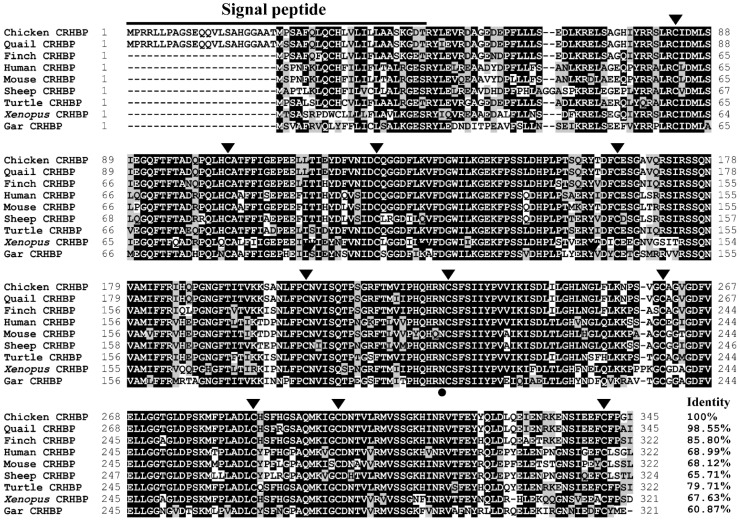
Amino acid sequence alignment of the chicken CRHBP precursor with that of Japanese quails (XP_015704133), zebra finches (XP_002190680), humans (NP_001873), mice (NP_940800), sheep (NP_001009339), Chinese soft-shelled turtles (XP_006119190), *Xenopus tropicalis* (XP_002940913) and spotted gars (XP_006626779). The horizontal line represents the signal peptides of the chicken CRHBP (46 amino acids). The ten conserved cysteine residues are marked with triangles. The location of N-linked glycosylation is marked with a circle. Identical amino acid residues are shown in black, whereas similar residues are labeled in gray. The identity of the CRHBP amino acid sequences between chickens and other vertebrate species was calculated and shown.

According to the predicted cDNA sequence of the chicken *CRHBP* deposited in the Ensembl Database (ENSGALT00010027397.1), we cloned the cDNA containing the complete open reading frame (ORF) of c*CRHBP* from the chicken brain tissue by RT-PCR. The cloned c*CRHBP* cDNA (accession no. OP259501) is 1038 bp in length and consists of 7 coding exons, which encodes a precursor protein with 345 amino acids (Figure 1). Sequence alignment revealed that the chicken CRHBP shares a high amino acid sequence identity with the CRHBP precursors from the Japanese quail (98.55%), zebra finch (85.80%), human (68.99%), mouse (68.12%), sheep (65.71%), Chinese soft-shelled turtle (79.71%), *Xenopus tropicalis* (67.63%) and spotted gar (60.87%). Furthermore, the ten cysteine residues, which form five disulfide bonds, and the N-linked glycosylation site were found to be conserved between chickens and other vertebrates (Figure 2). In addition, in the N-terminus of the chicken CRHBP, a putative signal peptide consisting of 46 amino acids was detected, which was longer than the signal peptides detected in mammals [16,18,22], reptiles [23] and fish [40,41].

**Figure 3:**
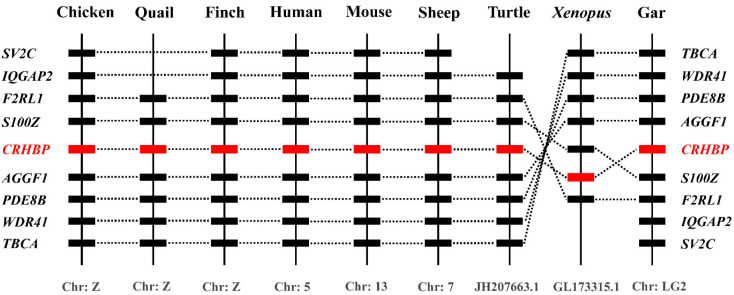
Synteny analyses of *CRHBP* in chickens and other vertebrates. *CRHBP* is localized in a distinct syntenic region conserved in chicken (*Gallus gallus*), quail (*Coturnix japonica*), finch (*Poephila guttata*), human (*Homo sapiens*), mouse (*Mus musculus*), sheep (*Ovis aries*), turtle (*Pelodiscus sinensis*), *Xenopus* (*Xenopus tropicalis*) and gar (*Lepisosteus oculatus*). Dotted lines indicate the syntenic genes identified in these species. Chr, chromosome.

Using synteny analysis, we found that *CRHBP* exists in chickens and other examined species, including humans (*Homo sapiens*), mice (*Mus musculus*), turtles (*Pelodiscus sinensis*), frogs (*Xenopus tropicalis*), quails (*Coturnix japonica*), finches (*Poephila guttata*), sheep (*Ovis aries*) and gars (*Lepisosteus oculatus*) (Figure 3). Moreover, some genes adjacent to *CRHBP* were consistent with those species examined, indicating that the chicken *CRHBP* was orthologous to the *CRHBP* genes of humans and other species.

### 3.2. Tissue Expression of CRHBP in Adult Chickens

**Figure 4:**
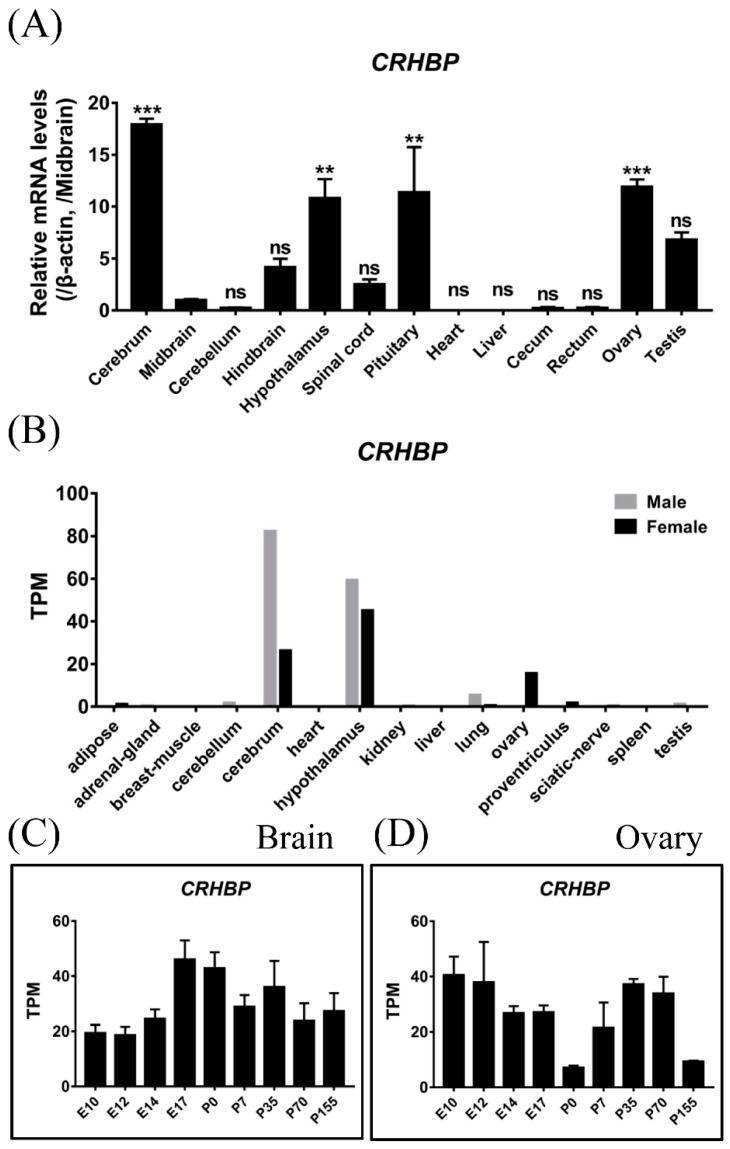
Expression of *CRHBP* in various chicken tissues. (**A**) Quantitative real-time PCR (qPCR) data showing the expression of *CRHBP* in adult chicken tissues. The mRNA level of *CRHBP* was normalized to that of *β-actin* and expressed as the fold difference compared with that of the midbrain. Each data point represents the mean ± SEM of 6 adult chickens (3 males and 3 females) (*n* = 6). **, *p* < 0.01 vs. midbrain; ***, *p* < 0.001 vs. midbrain; ns, no statistical difference vs. midbrain. (**B**) RNA-seq dataset from the red jungle fowl (SRP016501) showing the expression of *CRHBP* in various tissues. (**C**,**D**) RNA-seq dataset from red junglefowl (E-MTAB-6769) showing the expression of *CRHBP* in the brain (**C**) and ovary (**D**) during fetal (E10, E12, E14 and E17) and postnatal development stages (P0, P7, P35, P70 and P155). The transcripts per million (TPM) values were used to express the relative abundance of *CRHBP* transcripts.

Using qPCR, we examined the mRNA expression of *CRHBP* in adult chicken tissues. As shown in Figure 4A, the chicken *CRHBP* is abundantly expressed in the cerebrum, hypothalamus, pituitary and ovary, and weakly in the hindbrain, spinal cord and testis. In addition to qPCR, the two RNA-seq datasets deposited into the public database were also employed to analyze the expression pattern of *CRHBP*. According to the RNA-seq dataset from red jungle fowl (SRP016501), the expression of *CRHBP* mRNA was abundant in the cerebrum, hypothalamus and ovary (Figure 4B), consistent with our qPCR results. In another RNA-seq dataset from red jungle fowl (E-MTAB-6769), the expression of *CRHBP* mRNA showed an abundance in the brain (Figure 4C) and ovary (Figure 4D) during fetal (E10, E12, E14 and E17) and postnatal development stages (P0, P7, P35, P70 and P155).

### 3.3. Functional Analyses of Chicken CRHBP

Studies have shown that CRHBP binds CRH with nanomolar affinity, despite the structural difference between CRHBP and CRHR1 [14]. In addition, CRHBP inhibits CRH-mediated CRHR1 activation in HEK293T cells [15]. In the present study, the effect of cCRHBP on the signaling of CRHRs was examined in cultured CHO cells.

**Figure 5:**
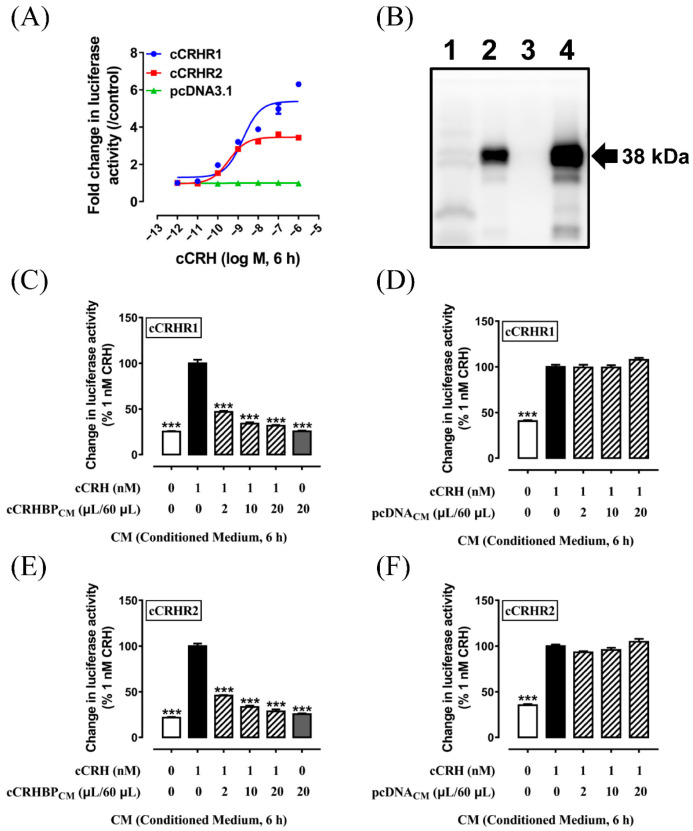
(**A**) Effect of cCRH on the signaling of cCRHR1 and cCRHR2 expressed in CHO cells. Each data point represents the mean ± SEM of triplicates (*n* = 3). (**B**) Detection of chicken His-tagged CRHBP by Western blot. Lane 1: cell lysate sample; Lane 2: cCRHBP conditioned medium before ultrafiltration; Lane 3: cCRHBP conditioned medium flow via ultrafiltration; Lane 4: cCRHBP conditioned medium after ultrafiltration. (**C**,**E**) Effect of cCRHBP conditional medium (cCRHBP_CM_) on the signaling of cCRHR1 (**C**) and cCRHR2 (**E**) activated by cCRH. (**D**,**F**) Effect of control conditional medium (pcDNA_CM_) on the signaling of cCRHR1 (**D**) and cCRHR2 (**F**) activated by cCRH. The luciferase activities were expressed as a percentage of the CRH treatment group. Each value represents the means ± SEM of four replicates (*n* = 4). ***, *p* < 0.001 vs. 1 nM CRH treatment control.

Using the pGL3–CRE–luciferase reporter system which was capable of monitoring receptor-stimulated cAMP-PKA signaling pathway, the effect of cCRH for the two CRH receptor subtypes (cCRHR1 and cCRHR2) transiently expressed in CHO cells were set in present study. As shown in Figure 5A, cCRH activates cCRHR1 (EC_50_: 1.73 nM) and cCRHR2 (EC_50_: 0.35 nM) with similar potencies. As a negative control, cCRH showed no influence on the luciferase activity of the CHO cells transfected with an empty pcDNA3.1(+) vector and a pGL3–CRE–luciferase reporter construct, supporting the effect of CRH peptide on the activation of CRHRs.

In the present study, the full length of *cCRHBP* cDNA was ligated into the expression plasmid, which was further transfected into HEK293 cells. The conditional medium from HEK293 cells with the cCRHBP–His expression plasmid was collected and concentrated. Western blot detected the band size of 38 kDa of chicken His-tagged CRHBP, which is in line with the report in humans [42], supporting that cCRHBP is a secreted protein (Figure 5B).

To determine the effect of the chicken CRHBP on the signaling of cCRHRs activated by cCRH, increasing amounts of cCRHBP_CM_ were added to the CHO cells that were transiently transfected with cCRHRs. As shown in Figure 5C,E, cCRHBP_CM_ (2–20 μL added, total volume 60 μL/well) was able to inhibit the signaling of cCRHR1 or cCRHR2 activated by cCRH (1 nM) in a dose-dependent manner. In contrast, the maximal volume of cCRHBP_CM_ (20 μL added, total volume 60 μL/well) showed no influence on the basal luciferase activities of cCRHR1 or cCRHR2 in CHO cells. As a negative control, as shown in Figure 5D,F, any amount of pcDNA_CM_ (2–20 μL added, total volume 60 μL/well) showed no effect on the signaling of cCRHR1 and cCRHR2. The present study supported that cCRHBP was able to efficiently inhibit the signaling of cCRHRs activated by CRH in CHO cells.

### 3.4. Effects of CRHBP on ACTH Secretion in Cultured Chick Pituitary Cells

**Figure 6:**
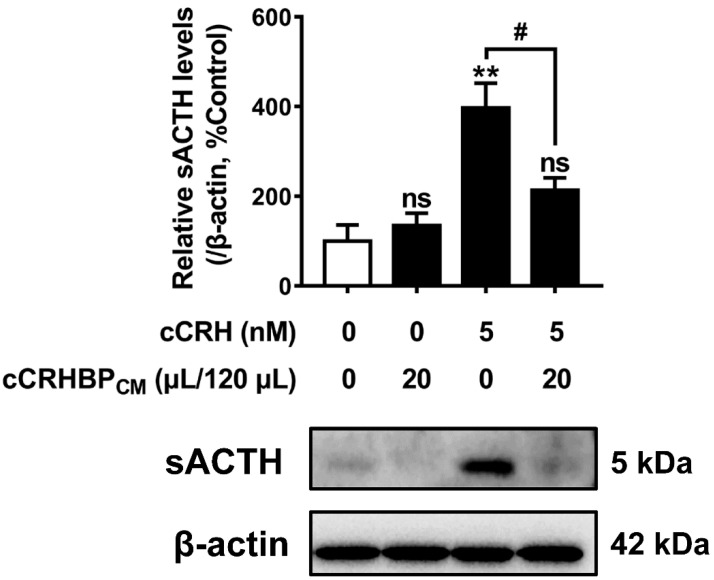
Effect of cCRHBP conditional medium (cCRHBP_CM_) on the ACTH secretion from cultured chick pituitary cells. The sACTH band at ∼5 kDa (secretory ACTH level detected in culture medium) was semiquantified by densitometric analysis. Their relative levels were normalized by that of β-actin in pituitary cell lysate and then expressed as a percentage of the control group. Each data point represents the mean ± SEM of 3 replicates (*n* = 3). **, *p* < 0.01, vs. control group (in the absence of cCRH and cCRHBP_CM_); ns, no statistical difference vs. control group; #, *p* < 0.05, vs. cCRH treatment group (in the absence of cCRHBP_CM_). Representative set of Western blots is shown at the bottom of each graph.

Given that *cCRHBP* was highly expressed in the chicken pituitary, and cCRHBP also inhibited the signaling of cCRHR1 activated by cCRH in CHO cells, the effect of cCRHBP on the pituitary ACTH secretion induced by cCRH was examined in the present study. As shown in Figure 6, cCRHBP_CM_ (20 μL added, total volume 120 μL/well) inhibited the ACTH secretion induced by cCRH (5 nM). Under similar conditions, the 20 μL cCRHBP_CM_ (total volume 120 μL/well) showed no effect on the baseline ACTH secretion.

### 3.5. The Expression Regulation of CRHBP mRNA by DEX and Stress in Chickens

In vertebrates, the ACTH secreted by the pituitary will further target the adrenal to promote glucocorticoid (GC) secretion, principally the corticosteroid (CORT) in chickens. To maintain the homeostasis of the HPA axis, GC could provide feedback on the hypothalamus and pituitary to inhibit CRH and ACTH synthesis/secretion. Whether cCRHBP participates in the feedback regulation was examined in the present study.

**Figure 7:**
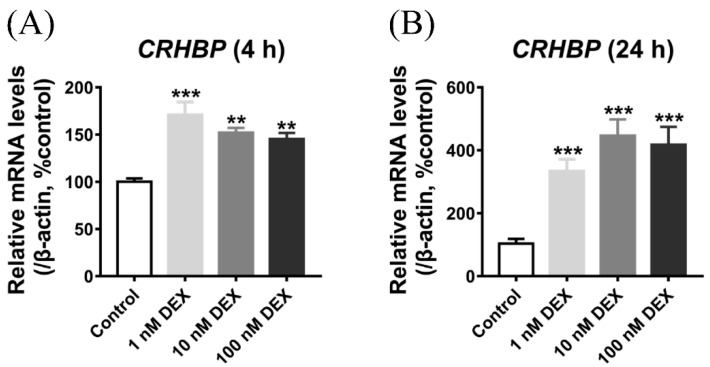
The effect of DEX (dexamethasone) on c*CRHBP* mRNA expression in cultured chick pituitary cells for 4 h treatment (**A**) and 24 h treatment (**B**). The mRNA levels were first calculated as the ration to that of *β-actin* and then expressed as a percentage of the control group. Each data point represents the mean ± SEM of 4 replicates (*n* = 4). **, *p* < 0.01 vs. control; ***, *p* < 0.001 vs. control.

In the cultured chick pituitary cells, the effect of DEX on *CRHBP* mRNA expression was first examined. As shown in Figure 7A,B, using qPCR, various doses of DEX (1, 10, 100 nM) significantly stimulated *CRHBP* mRNA expression after 4 h and 24 h treatment. Furthermore, the upregulation level of *CRHBP* mRNA expression in the 24 h after DEX treatment was significantly higher than that in the 4 h after DEX treatment.

**Figure 8:**
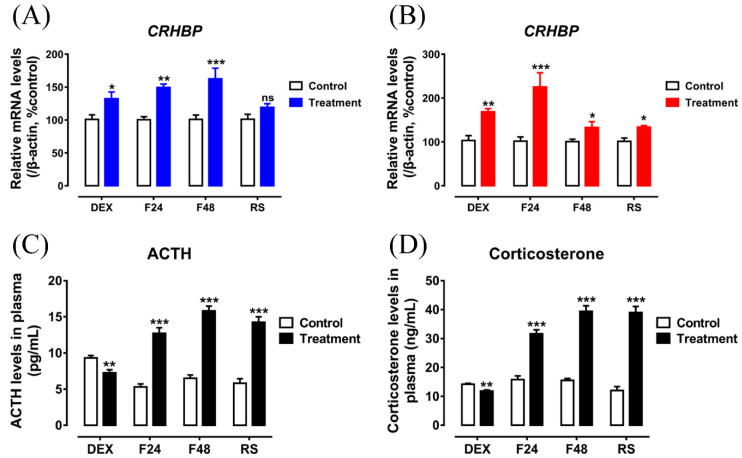
(**A**,**B**) The effects of different stress treatments on *cCRHBP* mRNA expression in the hypothalamus (**A**) and pituitary (**B**). The mRNA levels of *cCRHBP* were first calculated as the ration to that of *β-actin* and then expressed as a percentage of the control group. (**C**,**D**) The effects of different stress treatments on the concentration of ACTH (**C**) and corticosterone (**D**) in plasma. DEX: subcutaneous injection of dexamethasone. F24: fasting for 24 h. F48: fasting for 48 h. RS: restraint for 1 h. Each data point represents the mean ± SEM of 6 replicates (*n* = 6) *, *p* < 0.05 vs. control group; **, *p* < 0.01 vs. control group; ***, *p* < 0.001 vs. control group; ns, no statistical difference vs. control group.

In the present study, through in vivo studies, DEX was further injected subcutaneously to determine its effect on *CRHBP* mRNA expression in the chickens. As shown in Figure 8A,B, DEX (4 mg/kg body weight) substantially enhanced the expression of *CRHBP* in the hypothalamus and pituitary. In the present study, the concentrations of ACTH and corticosterone in plasma before or after the DEX injection were also detected. As shown in Figure 8C,D, DEX significantly decreased ACTH and corticosterone concentrations, supporting the feedback effect of DEX on the HPA axis.

Glucocorticoids, as the end products of the HPA axis to be stress-mediators, will be rapidly synthesized and secreted in response to the varied stressors [43]. Therefore, in the present study, the impact of stress on the *CRHBP* mRNA expression was further investigated. As shown in Figure 8A, the expression of *CRHBP* in the hypothalamus was significantly increased after 24 and 48 h of fasting, but its expression was steady after 1 h of restraint. Compared to the hypothalamus, the expression of *CRHBP* in the pituitary was considerably upregulated in response to all stress treatments (Figure 8B). Additionally, all three stress treatments significantly elevated the ACTH and corticosterone concentrations in the plasma (Figure 8C,D), supporting that the HPA axis was activated in response to the stress treatments.

## 4. Discussion

The present study, for the first time, characterizes the chicken *CRHBP* gene. Sequence analyses reveal that it shares a high sequence similarity with its counterparts in vertebrates, thus indicating its conserved role across species. Tissue expression analyses reveal that the *CRHBP* mRNA is abundantly expressed in the cerebrum, hypothalamus, pituitary and ovary. Functional assays demonstrate that CRHBP inhibits the signaling of cCRHRs activated by cCRH in CHO cells, thus reducing the cCRH-induced ACTH release in cultured chick pituitary cells. Moreover, stress-mediators (e.g., glucocorticoids) have been found to increase *CRHBP* mRNA expression in in vitro and in vivo studies. Together with the observation that stress stimulates *CRHBP* mRNA expression, our study supports the conserved role of cCRHBP as a negative feedback regulator in the chicken HPA axis.

### 4.1. CRHBP Is Conserved between Chickens and Other Vertebrate Species

In the present study, chicken *CRHBP*, which encodes a 345 amino acid precursor, was cloned for the first time (Figure 1). Sequence alignment showed that it shares high amino acid sequence identity with that of Japanese quails (98.55%), zebra finches (85.80%), humans (68.99%), mice (68.12%), sheep (65.71%), Chinese soft-shelled turtles (79.71%), *Xenopus tropicalis* (67.63%) and spotted gars (60.87%).

As shown in Figure 2, ten cysteine residues and one glycosylation site are conserved among the species examined, including chickens. The cysteine residues, which form five successive disulfide bonds, are essential for sustaining the biological activity of CRHBP [11]. Meanwhile, the asparagine N-linked-type oligosaccharides are reported to be essential for CRHBP binding [44]. In the present study, the amino acids (Arg68, Arg82, Arg79 and Asp85) have been detected to be conserved among the species (Figure 2). Using photoaffinity labeling experiments, the amino acid residues Arg46 and Arg59 of rat CRHBP are identified as the interaction sites with human/rat CRH_6–33_ [45]. Moreover, Huising et al. report that Arg56 and Asp62 of hCRHBP are required for binding to r/hCRH [15]. The essential amino acid conservation of chicken CRHBP with mammalian species suggests their similar binding features to their targets.

Additionally, synteny analysis supports that chicken *CRHBP* is orthologous to its counterparts in other vertebrates (Figure 3), indicating its conserved role across species.

### 4.2. CRHBP Is Widely Expressed in Chicken Tissues

In this study, qPCR and RNA-seq datasets were employed to detect the expression pattern of *CRHBP* in chicken tissues. Both the qPCR assay from chicken and the RNA-seq dataset from red jungle fowl demonstrate that *cCRHBP* mRNA is abundantly expressed in the central nervous system (CNS), pituitary and ovary. Within the CNS, *cCRHBP* shows a high expression level in the cerebrum and hypothalamus, which is accordant with the previous studies in rodents [26,28,46]. In the present study, in line with the reports in *Xenopus laevis* [23] and zebrafish [47], the *CRHBP* mRNA is also detected to be expressed in the brain. In addition, in the RNA-seq dataset from red jungle fowl (E-MTAB-6769), *CRHBP* has been found to be highly expressed at all developmental stages from fetal to postnatal (Figure 4C), suggesting that CBP-BP may play important roles in the chicken brain. In rodents, the *CRHBP* expressed in the brain, including the amygdala, bed nucleus of the stria terminalis (BNST), ventral tegmental area (VTA), prefrontal cortex (PFC) and various hypothalamic regions, is suggested to play key roles in stress-related disorders such as depression [27], anxiety [24], addiction [48] and Alzheimer’s disease [49].

In addition to the CNS, chicken *CRHBP* is also found to be abundantly expressed in the pituitary gland (Figure 4A). Our result is consistent with early reports that *CRHBP* is expressed in the rat and mouse pituitary [26,29,30]. Especially, the mouse *CRHBP* is detected to be highly expressed in the pituitary gland with a sexually dimorphic pattern, with females exhibiting much higher expression levels [30,50]. Single-cell sequencing from our research group further reveals that chicken *CRHBP* is abundantly expressed in pituitary folliculostellate cells (FS cells) [51], being different from the detection in female mice that *CRHBP* is expressed in pituitary cells, including corticotropes, lactotropes and gonadotropes [30]. The FS cells in the chicken pituitary are hypothesized to function as a paracrine/autocrine signaling hub, influencing neighboring cells [51]. Therefore, chicken CRHBP may function as a paracrine factor to regulate the wide range of pituitary functions, such as CRH-induced ACTH synthesis and secretion.

Interestingly, chicken *CRHBP* is also detected to be highly expressed in the ovary (Figure 4), suggesting its potential role in female chickens. The present study differs from a previous report in humans, in which *CRHBP* mRNA is not expressed in human ovarian follicles [52]. However, in rhesus monkeys, *CRHBP* mRNA is detected in theca cells of preovulatory follicles [53]. By binding plasma CRH, CRHBP may attenuate the hyperstimulation of the HPA axis caused by elevated placental CRH during pregnancy in humans [54,55]. In chickens, the *CRHBP* in the ovary may play a similar role in order to alleviate the stress response during egg production.

### 4.3. CRHBP Affects the Signaling of cCRHRs in CHO Cells

In the present study, using the pGL3–CRE–luciferase system, we demonstrated that cCRHBP_CM_ inhibited the signaling of cCRHRs activated by cCRH in a dose-dependent manner in CHO cells (Figure 5C). Our result is consistent with the finding in mammals, in which rCRHBP inhibits the activation of CRHR1 by 50 pM h/rCRH with an IC_50_ of 2.65 nM, as measured by a cAMP–luciferase reporter assay [15]. Present study is also consistent with the finding in common carp that recombinant CRHBP inhibits the r/hCRH-mediated CRHR1 activation [56].

In present study, cCRHBP_CM_ also inhibit the signaling of cCRHR2 activated by CRH (1 nM) in a dose-dependent manner (Figure 5E). Our result is partially similar to the finding in common carp, in which CRHBP inhibits the r/hCRH-mediated CRHR2s activation by cAMP-driven luciferase activity [56]. In nonmammalian species, including birds, CRH is also a potent thyrotropin (TSH)-releasing factor involving TSH expression and secretion through CRHR2 [57,58]. The present study supports the potential role of CRHBP involving chicken TSH synthesis and release.

Until the present, multiple functional mechanisms of CRHBP have been proposed, including the inhibition of CRHR1 [16,17,18], the promotion of CRHR2 [59,60], the independent of CRHRs [46,61] or as an escort protein of CRHR2α [62,63], which vary from tissues to species. The conserved amino acids (Arg68, Arg82, Arg79 and Asp85) detected in the chicken CRHBP revealed in the present study, together with the conserved motif (ARAE motif, Ala-Arg-Ala-Glu) detected in cCRH, with the report from the mammals that CRHBP binds CRH with nanomolar affinity [13,14,15,17], supports that chicken CRHBP may play an inhibitory function by decreasing the amount of ‘free CRH’, hence reducing the signaling of cCRHRs.

### 4.4. CRHBP Is a Negative Feedback Regulator in HPA Axis

Corticotropin (ACTH), as the critical part of the HPA axis, its biosynthesis and secretion from the pituitary gland, are controlled by the corticotropin-releasing hormone (CRH). In the present study, in chick pituitary cells, we demonstrated that cCRHBP_CM_ can inhibit CRH-induced ACTH secretion (Figure 6). Our result is consistent with the reports in mammals, in which CRHBP attenuates the CRH-induced ACTH secretion from primary anterior pituitary cells and AtT-20 cells [15,16,18]. Being the inhibitor of pituitary ACTH synthesis and release, the present study directly supports CRHBP as the negative feedback regulator in the HPA axis.

In the HPA axis, corticotropin (ACTH) will further target the adrenal to promote GC release to induce the serial cellular reactions [1,64]. As an essential negative feedback regulator of the HPA axis, glucocorticoids inhibit the expression of the pro-opiomelanocortin (POMC) gene in the pituitary gland, thus reducing ACTH synthesis [65,66]. In the present study, we found that DEX may significantly increase the expression of *CRHBP* mRNA in cultured chick pituitary cells (Figure 7A, B). In addition, glucocorticoids also positively upregulated the pituitary *CRHBP* mRNA expression with a single subcutaneous injection of DEX (4 mg/kg body weight) (Figure 8B). Consequently, in chickens, both in vitro and in vivo findings support that the expression of *CRHBP* mRNA is upregulated in the pituitary gland under GC treatment. In addition to the pituitary, hypothalamic *CRHBP* mRNA expression can also be upregulated by a subcutaneous injection of DEX (Figure 8A). As the inhibitor for ACTH synthesis and secretion, the elevated *CRHBP* expression in the hypothalamus–pituitary axis in response to the DEX treatment will further inhibit ACTH release, thus supporting its role as a negative feedback inhibitor in the HPA axis. The present study is in line with the detection in mammals [11,12]. In addition, our result is also similar with the finding in rats, in which adrenalectomy reduces pituitary *CRHBP* mRNA expression to 8% of sham adrenalectomy rats [67].

In vertebrates, as the most important neuroendocrine system in response to stress, the HPA axis will be activated in response with many kinds of stressors, including temperature and starvation [43,68,69]. In the present study, the effect of different stress treatments on the expression of *CRHBP* was further investigated. Both fasting and restraint treatments could significantly increase the expression of *CRHBP* in the pituitary (Figure 8B), thus being in line with the detection in rats that restraint stress significantly increases the pituitary *CRHBP* mRNA levels from 30 min to 2 h [67]. The present study is also in line with the observance in mice that restraint stress elevates the pituitary *CRHBP* mRNA levels, particularly in the female pituitary [50]. In addition to the pituitary, fasting treatment also positively upregulates the expression of *CRHBP* in the chicken hypothalamus (Figure 8A). The hypothalamus, as the convergence center from various nuclei and nerve fibers, plays vital physiological functions, including stress, reproduction, energy metabolism and thermoregulation [70]. The present study is in accordance with the report from the fish and *Xenopus laevis* that the hypothalamic *CRHBP* expression is regulated by stress [23,40,41]. In present study, the ACTH and corticosterone concentrations in the plasma were detected to be upregulated, revealing the activation of the HPA axis in response to various stress treatments. The upregulation of *CRHBP* mRNA expression is thus most likely regulated via enhanced stress mediators (e.g., glucocorticoid) release. The stress-induced upregulation of *CRHBP* mRNA may further augment the glucocorticoid-mediated feedback inhibition of the HPA axis, thus supporting its role as a negative feedback regulator in the HPA axis.

In summary, the gene information and tissue expression of *CRHBP* have been investigated in chickens for the first time. Functional assays prove that chicken CRHBP inhibits the signaling of cCRHR1 (or cCRHR2) activated by cCRH in CHO cells, which further reduces the ACTH secretion in chick pituitary cells. In combination with the observance that glucocorticoids and stress significantly stimulate *CRHBP* mRNA expression in chickens, the present study supports that CRHBP may function as a negative feedback regulator along the HPA axis, possibly by modulating the CRH–CRHR1 signaling pathway. As an important poultry animal, the growth, reproduction, disease resistance, etc., of chickens are severely impacted by the HPA axis [71,72,73,74]. The characterization of the chicken *CRHBP* gene helps to reveal the molecular basis of the chicken HPA axis and is thus beneficial to the poultry industry.

## Figures and Tables

**Table 1:** Primers used in this study ^a^.

Gene/Construct Name	Sense/ Antisense	Primer Sequence (5′–3′)	Size (bp)	Accession Number
Primers used for cloning of CDS ^b^				
*CRHBP*	Sense	CCCAAGCTTCTCCCAACTGCTCTATA	1071	OP259501
	Antisense	CGGGGTACCTCAGATACCAGGGAAGC		
Primers used for quantitative real-time RT-PCR assay				
*CRHBP*	Sense	CGCCACATTCTTCATCGGTG	216	OP259501
	Antisense	GATGACCTGATGCTCCTCTG		
*β-actin*	Sense	CCCAGACATCAGGGTGTGATG	123	L08165.1
	Antisense	GTTGGTGACAATACCGTGTTCAAT		
Primers for construction of the expression plasmids ^b^				
*CRHBP-His*	Sense	AGTCCAGTGTGGTGGAATTCCTGAAGATGCCCCGGCGGCT	1056	
	Antisense	TCAATGATGATGATGATGATGGATACCAGGGAAGCAGAAT		
	Antisense	CTGGATATCTGCAGAATTCTCAATGATGATGATGATGATG		

^a^ All primers were synthesized by Youkang Biotechnology (Chengdu, China). ^b^ Restriction sites added at the 5’-end of the primers are underlined.

## Data Availability

Not applicable.
